# Symptoms of anxiety/depression during the COVID-19 pandemic and associated lockdown in the community: longitudinal data from the TEMPO cohort in France

**DOI:** 10.1192/j.eurpsy.2022.361

**Published:** 2022-09-01

**Authors:** A.J. Andersen, M. Melchior, M. Mary-Krause, J.J. Herranz Bustamante, T. El Aarbaoui, M. Héron

**Affiliations:** INSERM, Sorbonne University, Team Of Social Epidemiology (eres), Pierre Louis Institute Of Epidemiology And Public Health (iplesp), Paris, France

**Keywords:** Symptoms of anxiety/depression, Longitudinal study, lockdown, Covid-19

## Abstract

**Introduction:**

The COVID-19 pandemic and associated preventive measures have an impact on the persons’ mental health, including increasing risk of symptoms of anxiety and depression in particular. Individual experiencing mental health difficulties in the past could be especially vulnerable during lockdown, however, few studies have tested this empirically considering preexisting mental health difficulties using longitudinal data.

**Objectives:**

The objective of this study is to examine the longitudinal association between preexisting symptoms of anxiety/depression and symptoms of anxiety/depression during lockdown due to the COVID-19 pandemic in a community sample.

**Methods:**

Seven waves of data collection were implemented from March-May 2020. Generalized estimation equations models were used to estimate the association between preexisting symptoms of anxiety/depression and symptoms of anxiety/depression during lockdown among 662 mid-aged individuals from the French TEMPO cohort.

**Results:**

We found an elevated odds ratio of symptoms of anxiety/depression (OR=6.73 95% [CI=4.45–10.17]) among individuals experiencing such symptoms prior lockdown. Furthermore, the odds of symptoms of anxiety/depression during lockdown was elevated among women (OR=2.07 [95% CI=1.32–3.25]), subjects with low household income (OR=2.28 [1.29–4.01]) and persons who reported loneliness (OR=3.94 [2.47–6.28]).

**Conclusions:**

This study demonstrates a strong relationship between preexisting symptoms of anxiety/depression and anxiety/ depression during the COVID-19 outbreak among mid-aged French adults. The findings underline the role of preexisting symptoms of anxiety/depression as a vulnerability factor of anxiety/depression during lockdown. Furthermore, the study shows that loneliness is independently associated with symptoms of anxious/depression, when controlling for prior anxiety/depression symptoms.

**Disclosure:**

No significant relationships.

